# Supplementary material for: Anomalous friction of supercooled glycerol on mica
Source: Nat Commun. 2024 Jul 20;15:6129. doi: 10.1038/s41467-024-50232-0 (PMC11271443; doi:10.1038/s41467-024-50232-0)
Supplement: Supplementary file 1 — Supplementary Information [file 41467_2024_50232_MOESM1_ESM.pdf]

Anomalous friction of supercooled glycerol on mica
SUPPLEMENTARY INFORMATION

Mathieu Lizée,[1,*] Baptiste Coquinot,[1] Guilhem Mariette,[1] Alessandro Siria,[1] and Lydéric Bocquet[*1]

[1]*Laboratoire de Physique de l'Ecole Normale Supérieure 75005 Paris France*

I. SUPPLEMENTARY METHODS

A. Glycerol's bulk dynamical properties

Our analysis of the interfacial friction coefficient $\lambda = \eta/b$ requires a good knowledge of glycerol's viscosity as a function of temperature and at 30 kHz. In the following, we describe how bulk viscosity and relaxation dynamics are estimated.

1. *Viscosity measurement*

We measure the viscosity of our glycerol's sample with a cone-plane rheometer and plot it as a thick blue line on Figure S1**a**. Then, we use the interpolation formula $\eta(c_{water}, T)$ proposed by Ref.[1] for which the best fit (black solid line) is obtained with a small water content of 0.5%. Whereas the rheology measurement was performed in room conditions, the AFM measurements take place in a vacuum chamber on a millimetric glycerol droplet which remained at mbar pressure for several days. We thus view this 0.5% water contamination level as an upper bound. Considering the tiny effect this would have on viscosity curves, possible effects of water contamination on bulk rheology are neglected in the rest of the study.

Shear modulus The dashed blue lines on Figure S1**a** are the real and imaginary parts of the 30 kHz shear viscosity defined as $\eta'(\omega) - i\eta''(\omega) = G(\omega)/i\omega$ for pure glycerol from the Barlow-Erginsav-Lamb (BEL) model. The BEL model introduced in Ref.[2] is an empirical equation used to describe the frequency-dependent shear mechanical impedance of supercooled liquids. In this model, the complex modulus $G(\omega) = \sigma(i\omega)/\gamma(i\omega)$ (σ and γ are the shear stress and strain) is given as a function of the temperature-dependent limiting shear modulus G_∞ and the static viscosity $\eta(T)$.

$$G(\omega)^{-1} = \frac{1}{G_\infty} + \frac{1}{i\eta\omega} + 2\Big(\frac{1}{i\eta\omega G_\infty}\Big)^{1/2} \tag{1}$$

Whereas η varies exponentially with temperature, G_∞ typically shows an inverse dependency on absolute temperature :

$$\frac{1}{G_\infty} = \frac{1}{G_0} + c(T - T_0) \tag{2}$$

where $T_0 = -87°\mathrm{C}$, $G_0 = 5$ GPa and $c = 18 \times 10^4$ GPa^{-1}.K^{-1} (from Ref.[3]). From the complex shear modulus, one easily recovers the complex bulk viscosity : $\eta'(\omega) - i\eta''(\omega) = G(\omega)/i\omega$ which we plot as a function of temperature on Figure S1**a**. In our analysis, we verified that taking into account the small visco-elasticity of glycerol (non-zero η''(T,30kHz)) leaves all results unchanged. Indeed, all our experiments verify $\eta''(30\mathrm{kHz})/\eta'(30\mathrm{kHz}) < 30$ and are thus deep in the newtonian regime.

2. *Broadband dielectric spectroscopy*

To estimate glycerol's friction on mica from Green-Kubo equation (see the main text), we need glycerol's density structure factor $S_l(q, \omega)$. As a fluctuation correlator, S_l can be estimated from absorption measurements, using again the Green-Kubo relations. Mechanical spectroscopy is ideal to estimate the *density*

* mathieu.lizee@ens.fr, lyderic.bocquet@ens.fr

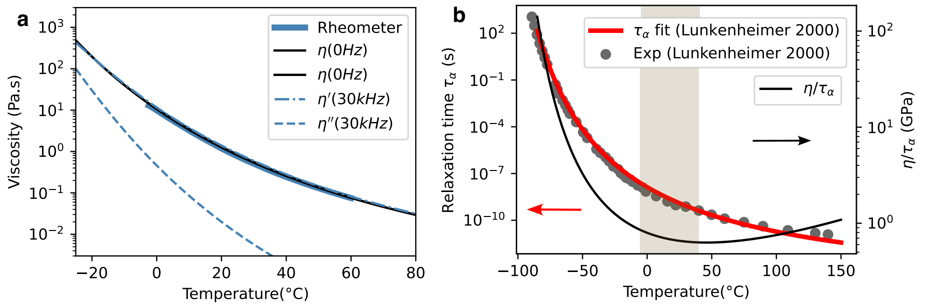

FIG. S1: **a** Glycerol viscosity measured with a rheometer along with the fit with interpolation formula from Ref.[1] (black). Blue lines are the complex viscosity at 30 kHz determined using the BEL model described in [4]. **b** Dielectric relaxation time (see Figure S2**b**) and Maxwell product η/τ_α *versus* frequency. The shaded area denotes the experimentally accessed temperature range. Source data are provided as a Source Data file.

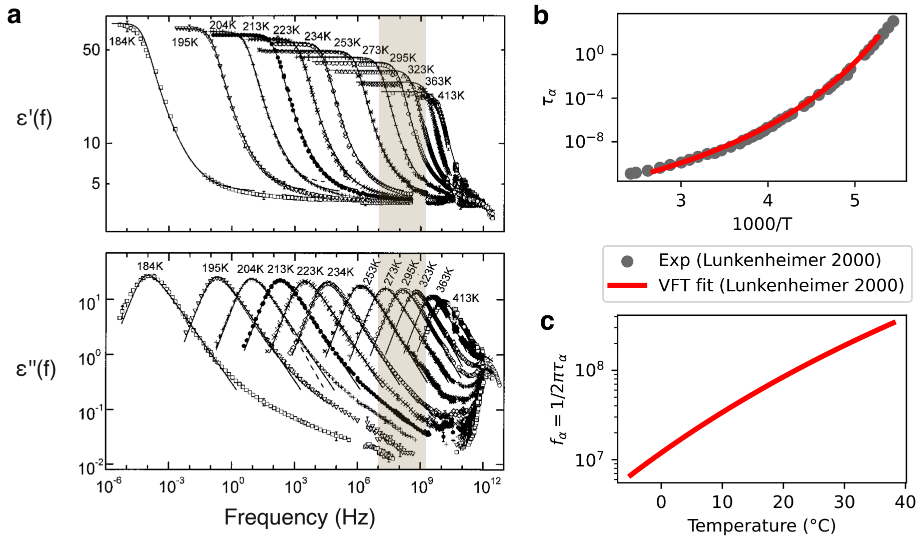

FIG. S2: **a** Experimental measurements of broadband dielectric spectroscopy reproduced from Ref. [6]. The shaded area denotes the experimentally accessed temperature range **b**. Dielectric relaxation time defined as the inverse of the dielectric absorption peak frequency f_α *versus* inverse temperature for pure glycerol. This Arrhenius plot shows a non-linear dependency of $\log(\tau)$ on inverse temperature, a feature of glycerol's fragility. **c** Peak frequency f_α as a function of temperature on the relevant temperature range for our experimental study.

structure factor. Unfortunately, although detailed shear rheology analysis were conducted on glycerol [3, 5], they use shear wave spectroscopy, limited to $f < 30$ MHz. This frequency regime being very low in front of typical solid state dynamics, it is not relevant to evaluate dynamic contributions to liquid-solid friction. Luckily, the polar nature of glycerol allows us to track structural re-arrangements through their dielectric permittivity $\epsilon(i\omega)$ whose frequency dependency is similar to that of the the inverse shear impedance $1/G(\omega)$. Thus, we leveraging broadband dielectric spectroscopy measurements over a large temperature range to capture glycerol's dynamics in the MHz-Hz region.

We display on Figure S2**a** experimental broadband dielectric spectra of glycerol reproduced from Ref.[6]. The main α relaxation peak can be described by the Cole-Davidson ansatz introduced in 1951 in Ref. [7] to describe the broad distribution of relaxation timescales in glycerol and propylene glycol:

$$\epsilon(\nu) = \epsilon_\infty + \frac{\epsilon_S - \epsilon_\infty}{(1 + 2i\pi\omega/\omega_\alpha)^\beta}. \quad (3)$$

with $\beta \sim 0.65$, slowly varying with temperature [6, 8]. The dielectric response is peaked at the characteristic relaxation frequency $f_\alpha = \omega_\alpha/2\pi$ which we identify to the liquid's relaxation rate. ω_α follows the Vogel-

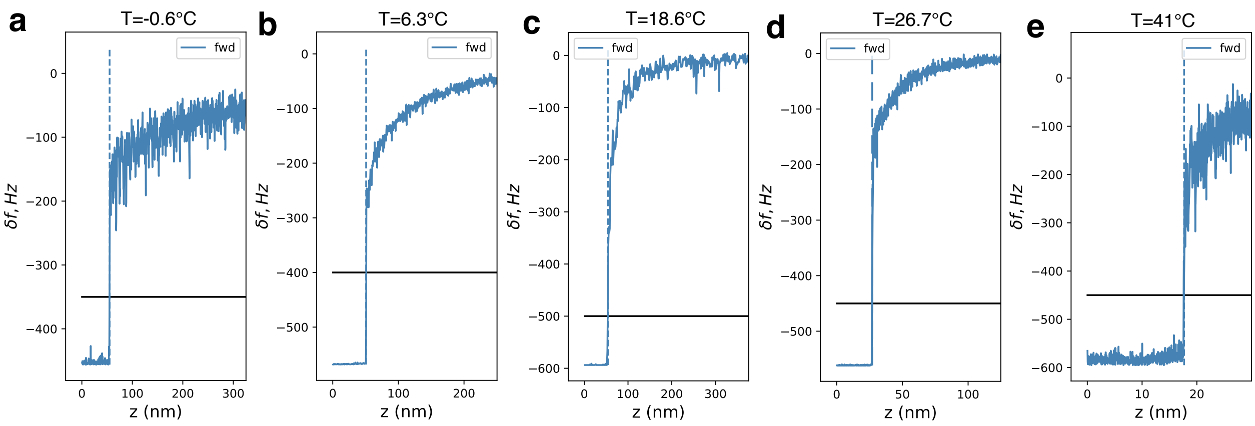

FIG. S3: **Solid contact detection** for an oscillation amplitude of 3 nm and temperatures ranging from -0.6 to 41°C on panels **a-e**. The black line denotes a threshold frequency shift which is chosen independently for each curve in order to optimize the precision on the contact's determination.

Fulcher-Tammann law with parameters given by Ref.[6]. We have :

$$\tau(T) = \tau_0 \exp\left(\frac{DT_{\mathrm{VF}}}{T - T_{\mathrm{VF}}}\right) \tag{4}$$

with $T_{\mathrm{VF}} = 129\mathrm{C} = -144°\mathrm{C}$, $D = 17.9$ and $f_0 = 1.1 \times 10^{14}$ Hz (note that $\tau = 1/2\pi f_\alpha$). On Figure S2**b**, we show the Arrhenius plot of glycerol's relaxation time whose non linearity signals the fragile character. A closer look on Figure S2**c** shows that glycerol's relaxation rate varies from 10 to 300 MHz on the temperature range addressed in our experiments. Finally, we plot on Figure S1**b** the dielectric relaxation time τ_α (red) as well as the product $\eta\omega_\alpha$ in GPa. Clearly, this 'Maxwell modulus' is roughly constant in the experimentally adressed temperature range, suggesting that a single relaxation time dominates structural rearrangements and that a Maxwell model of viscoelasticity is valid for glycerol in this temperature range. One should take this argument with a pinch of salt as the dielectric α peak is described by a Davidson-Cole equation with $\beta \neq 1$ implying a distribution of relaxation timescales [4, 7].

B. Hydrodynamics of the drainage flow

Our slip length measurements rely on the measurement of viscous dissipation as a tungsten tip, approaches the planar mica surface.

1. *Solid contact detection*

We here provide more details on the solid contact's detection at various temperatures. As described in the main text, we use a sudden drop in the frequency shift signal δf to determine the position of the sphere-plane contact with nanometric precision. We show the frequency shift approach curves for various temperatures and the corresponding solid contact positions on Figure S3.

2. Influence of the oscillation's amplitude We now shopw that the tuning fork's oscillaiton amplitude has little effect on the measurement. On Figure S4, we plot several curves obtained at 25° C from the same dataset as presented in the main text. Here, we show that for oscillation amplitudes a ranging from 1 to 5 nm, the measured slip length remains constant (b= 3.5 ±1nm here).

3. *Fitting the approach curves*

The experimental approach curves were fitted with the analytical expressions derived by Vinogradova [9] in the assumption that slippage only occurs on one surface. They allow to fit the curves with an excellent precision down to very small distance. The quality of the fits in the curved regions (*cf.* Figure 2 of the main

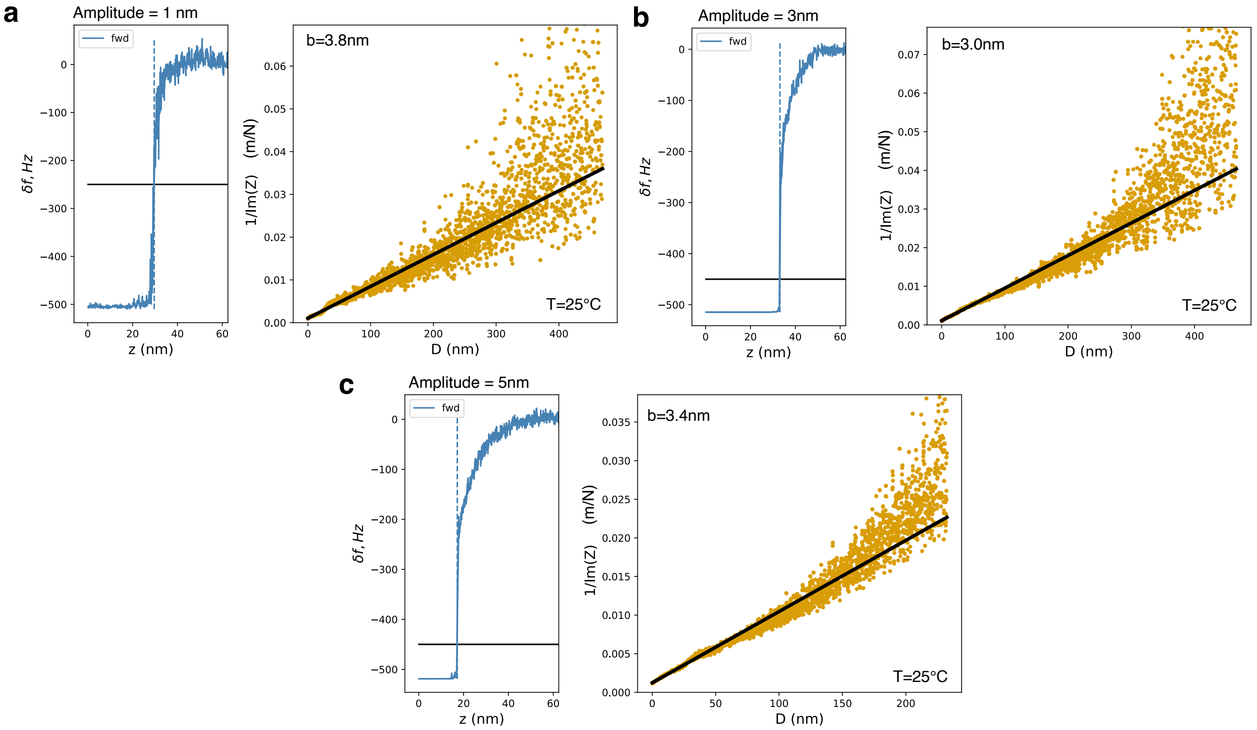

FIG. S4: **Dependency of approach curves on the tuning fork's oscillation amplitude at 25**$^\circ C$ We plot the frequency shift δf with the corresponding solid contact's position with blue curves and the inverse dissipative impedance $1/Im(Z)$ with golden points. The black lines are fits with the Reynolds equation. Panels **a-c** correspond respectively to oscillation amplitudes of 1, 3 and 5 nm.

text) brings considerable control to the slippage measurements. It was a similar fitting process that allowed dynamic surface force apparatus (d-SFA) experiments to quantitatively account for slippage on hydrophobic surfaces [10]. Writing $y = b/D$ and assuming a no slip boundary condition on the tungsten tip, the function f^* in Eq.3 of the main is:

$$f^*(\tilde{y}) = \frac{1}{4}\left(1 + \frac{3}{2\tilde{y}}\left[\left(1 + \frac{1}{4\tilde{y}}\right)\log(1 + 4\tilde{y}) - 1\right]\right) \tag{5}$$

a. Fitting process In practice, the energy dissipation E at a distance D is expressed in units of its far-field value E_0:

$$\frac{E(D)}{E_0} = 1 + \mathfrak{Im}\left[\frac{\eta \times \alpha}{D + h_0} f^*\left(\frac{b}{D + h_0}\right)\right] \tag{6}$$

where α, E_0, and b are adjusted to fit the distance-dependency. Finally, the shift distance h_0 accounts for the residual roughness of the tungsten tip (*ie,* the distance between solid contact and the mean sphere surface). Here h_0 is measured as the residual apparent total slip length at high temperature with the Reynolds formula leading to $h_0 = 10\pm2$nm. This value of h_0 is subsequently fixed for all temperatures. E_0 and α are adjustable parameters but their values are strongly constrained due to their clear physical meaning: E_0 is the dissipation far from the surface and α is an instrumental factor which is estimated as

$$\alpha \equiv \frac{12\pi^2 R^2 f_0 Q_0}{K_{\text{eff}}}.$$

In this formula, the tip's radius $R = 6.5\mu$m is measured from electron microscope images (*cf.* Figure 1**a** of the main text) while $f_0 = 30$ kHz and $K_{\text{eff}} \simeq 40$ kN/m. This direct analysis of dissipation curves $E(D)$ is both a robust and well-controlled way of measuring the friction coefficient of glycerol on mica $\tilde{\lambda}(T)$ as a function of temperature.

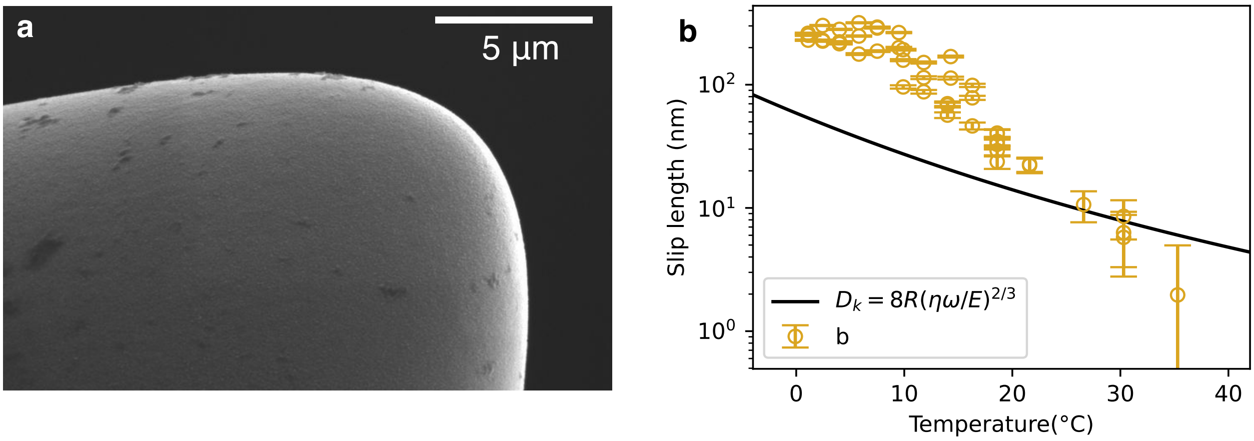

FIG. S5: **a** SEM image of the tungsten tip used in this paper. On panel **b**, we plot the elasto-hydrodynamic length *versus* temperature compared with the slip length. The EHD critical distance is clearly much smaller than b for T<30°C.

II. SUPPLEMENTARY DISCUSSION

1. *Dynamic AFM hydrodynamics*

Although the dynamic AFM technique has an exquisite sensitivity to the solid contact position compared to the usual static cantilever AFM, the hydrodynamic flow analysis tedious and requires to take care of several effects such as visco-elasticity, elasto-hydrodynamics and inertial drag. On Figure S1, we have shows that the imaginary part of glycerol's viscosity can be neglected in our temperature range. In the following, we show that in our experiments, both these effects can be safely neglected.

2. *Elasto-hydrodynamic correction*

At high frequency and high viscosity, it is well known that sphere-plane drainage may lead to an elastic deformation of solid surfaces. A correstion on the dissipative impedance has to be included when solid deformation is strong [11] leading to a saturation of Z'' before the true solid contact. In our data, we never observed such saturation and thus believe elastic deformations of mica to be negligible. Still, we show it quantitatively with a simple comparison : the so-called elasto-hydrodynamic correction is strong for confinement lower than $D_k = 8R(\eta\omega/E)^{2/3}$ where $E \sim 60$ GPa is the out-of-plane Young modulus of mica [12]. This critical elasto-hydrodynamic length D_k increases at low temperature with viscosity. We show on Figure S5**b** that D_c is lower than the slip length except for the very hottest conditions yielding negligible surface deformations. Mica's deformation is thus believed to be extremely small and has no effect on force distance curves and their analysis.

As a final note, while the glue – silver paste – is softer than mica and could possibly lead to an enhanced elasto-hydrodynamic response, this effect is expected to be negligible. Indeed, the surface of the glued part is larger than the hydrodynamic area by a factor 10^5 : $S_{glue} \sim 10^{-8}$ m^2 $\sim 10^5 Rh$. In this context, the elasto-hydrodynamic response of the glue is completely negligible as compared to that of the tip and substrate.

3. *Shear-rate dependency*

Several studies have reported non linearities in the slippage process, namely a dependency of slip length on the shea-rate $\dot{\gamma} = \partial_z v_x$. In our experiment, we ensure that the drainage flow is in the linear regime $\dot{\gamma} \sim fa/D \ll \omega_\alpha$.

4. Inertia effects

To safely neglect inertia effects, we follow Ref.[13] and compute the Womersley number of our experiment $W_0 = R\sqrt{\omega\rho/\eta} < 5.10^{-2} \ll 1$. In this low W_0 regime, inertia has no effects on the dissipation approach curves $Z''(D)$ and we can safely neglect it in our analysis.

A. Interfacial friction of glycerol : theoretical estimate

1. Green-Kubo formula

The total friction coefficient λ can be expressed through the Green-Kubo formula:

$$\lambda = \frac{1}{k_\mathrm{B}T}\frac{1}{\mathcal{A}}\int_0^\infty \mathrm{d}t\,\langle \mathbf{F}_x(t)\mathbf{F}_x(0)\rangle \tag{7}$$

where $\mathbf{F}$ is the force between the solid and the liquid and the average is taken at equilibrium. Here, the mica has no electronic modes thus the force is made wholly of the repulsion between the solid's and liquid's atoms. Denoting V the repulsion potential between the atoms of the solid and the liquid, the force writes

$$\mathbf{F}(t) = \int \mathrm{d}\mathbf{r}_\ell \mathrm{d}\mathbf{r}_\mathrm{s}\, n_\ell(\mathbf{r}_\ell, t)\nabla V(\mathbf{r}_\ell - \mathbf{r}_\mathrm{s}) n_\mathrm{s}(\mathbf{r}_\mathrm{s}, t) \tag{8}$$

with n_ℓ the particle density of the liquid and n_s of the solid.

The repulsion only concern the closest atoms and thus we can restrict the integrations over the first layer of atoms of both the solid and the liquid. We denote z the distance between these two layers. Moreover, at first order, the fluctuations of the solid's and liquid's densities are uncorrelated. Thus, the relevant input for the Green-Kubo formula is the structure factor of the first layer

$$S_\alpha(\mathbf{r}, t, \mathbf{r}', t') = \langle n_\alpha(\mathbf{r}, t) n_\alpha(\mathbf{r}', t')\rangle \tag{9}$$

This is a function of $\mathbf{r} - \mathbf{r}'$ (parallel to the surface) and $t - t'$ by translation invariance. Finally, the friction coefficient becomes

$$\lambda = \frac{1}{2\mathcal{A}k_\mathrm{B}T}\int \mathrm{d}t\mathrm{d}\mathbf{r}_\ell^1\mathrm{d}\mathbf{r}_\mathrm{s}^1\mathbf{r}_\ell^2\mathrm{d}\mathbf{r}_\mathrm{s}^2\, \nabla_x V(\mathbf{r}_\ell^1 - \mathbf{r}_\mathrm{s}^1) S_\ell(\mathbf{r}_\ell^1 - \mathbf{r}_\ell^2, t)\nabla_x V(\mathbf{r}_\ell^2 - \mathbf{r}_\mathrm{s}^2) S_\mathrm{s}(\mathbf{r}_\mathrm{s}^1 - \mathbf{r}_\mathrm{s}^2, t). \tag{10}$$

Going to Fourier space, we obtain,

$$\boxed{\lambda = \frac{1}{8\pi^2 k_\mathrm{B}T}\int_0^\infty \mathrm{d}\omega\mathrm{d}q\, q^3 V(q,z)^2 S_\ell(q,\omega) S_\mathrm{s}(q,\omega)} \tag{11}$$

which is the general formula for the friction coefficient.

In the following, we estimate the liquid and solid's structure factors S_ℓ and S_s to allow a quantitative estimate of Eq.11.

2. Solid's dynamical structure

We now turn to the solid. The latter's structure factor can be decomposed in two parts: a static contribution related to the surface roughness and a dynamic contribution related to the internal mechanical degrees of freedom of the solid, typically the phonons. Thus,

$$S_\mathrm{s}(\mathbf{r}, t, \mathbf{r}', t') = S_\mathrm{s}^\mathrm{stat}(\mathbf{r}, \mathbf{r}') + S_\mathrm{s}^\mathrm{dyn}(\mathbf{r}, t, \mathbf{r}', t') \tag{12}$$

which, becomes in Fourier space

$$S_\mathrm{s}(\mathbf{q}, \omega) = S_\mathrm{s}^\mathrm{stat}(\mathbf{q})\delta(\omega) + S_\mathrm{s}^\mathrm{dyn}(\mathbf{q}, \omega). \tag{13}$$

As a consequence, the friction coefficient also divides into two parts: the static and the dynamic contributions to the friction:

$$\lambda = \lambda_\mathrm{stat} + \lambda_\mathrm{dyn} \tag{14}$$

where

$$\lambda_{\rm stat} = \frac{1}{8\pi^2 k_{\rm B}T}\int_0^\infty {\rm d}q\, q^3 V(q,z)^2 S_\ell(q,\omega=0) S_{\rm s}^{\rm stat}(q) \tag{15}$$

and

$$\lambda_{\rm dyn} = \frac{1}{8\pi^2 k_{\rm B}T}\int_0^\infty {\rm d}\omega {\rm d}q\, q^3 V(q,z)^2 S_\ell(q,\omega) S_{\rm s}^{\rm dyn}(q,\omega) \tag{16}$$

Overall, the solid's structure factor shows two main features at zero and high frequency which we sketched on Figure 4 of the main text (black solid line). These two features lead us to define two complementary contributions to friction. Firstly, the static contribution involves the liquid's static structure factor in the $\omega \ll \omega_\alpha$ and the solid's corrugation landscape. Moving towards high frequency modes, the dynamic contribution builds up, based on the overlap of liquid and solid's structure factors in the (q,ω) space.

3. Liquid's dynamical structure

Now turning to glycerol's structure factor S_ℓ, we recall the Cole-Davidson shape of the dielectric relaxation (Eq.S2) and its characteristic triangular shape in log-log representation (*cf.* Figure S2). In analogy to the dielectric response, we assume that the mechanical response has such a shape and use a slightly simpler ansatz for the dynamical structure factor : a Debye like model centered at $\omega_\alpha = 2\pi f_\alpha$ with spatial structure $\phi(q)$ (*cf.* Figure S2).

$$\chi(\mathbf{q},\omega) \approx \frac{\phi(q)}{1 - i\omega/\omega_\alpha} \tag{17}$$

The fluctuation-dissipation theorem then states that

$$S_\ell(q,\omega) = \frac{2k_{\rm B}T}{\omega}{\rm Im}\left[\chi(\mathbf{q},\omega)\right] = 2\phi(q)k_{\rm B}T\frac{\omega_\alpha}{\omega_\alpha^2+\omega^2}. \tag{18}$$

In the low frequency limit, the structure factor saturates to a constant value fixed by the α peak frequency f_α

$$S_\ell(q,\omega) \approx \frac{2\phi(q)k_{\rm B}T}{\omega_\alpha} \propto 1/\omega_\alpha \tag{19}$$

which is consistent with a diffusion process of coefficient $D \propto \omega_\alpha$. Indeed, we expect that at small frequencies, the structure factor of a liquid is dominated by the diffusion of molecules with a coefficient D: $S_\ell(q,\omega) \sim 1/Dq^2$ leading to a simple scaling of the static friction coefficient $\lambda_{\rm stat} \propto 1/{\rm D}$ [14].

In the high frequency region however, the liquid's structure factor scales with

$$S_\ell(q,\omega) \approx \frac{2\phi(q)k_{\rm B}T\omega_\alpha}{\omega^2} \propto \omega_\alpha \tag{20}$$

and strongly depends on ω. It is interesting to note that the liquid's structure factor itself has the same scaling with ω_α as the experimental friction coefficient $\lambda(f_\alpha)$ (see Figure 4 of the main text) in both the low and high frequency limits.

Supercooled liquid's friction on a solid's surface From the previous analysis, we write the general formulae for the friction of a supercooled liquid on an arbitrary solid surface as a function of its relaxation rate $\omega_\alpha/2\pi$.

Injecting the low frequency liquid's structure factor in Eq.15 yields:

$$\boxed{\lambda_{\rm stat} = \frac{1}{4\pi^2\omega_\alpha}\int_0^\infty {\rm d}q\, q^3 V(q,z)^2 S_{\rm s}^{\rm stat}(q,\omega)\phi(q)} \tag{21}$$

Likewise, in the usual case where solid state dynamics modes are much faster than the liquid's α peak, we obtain from Eq.16 that the dynamical friction simply writes:

$$\boxed{\lambda_{\rm dyn} = \frac{\omega_\alpha}{4\pi^2}\int_0^\infty {\rm d}\omega{\rm d}q\, q^3 V(q,z)^2 S_{\rm s}^{\rm dyn}(q,\omega)\frac{\phi(q)}{\omega^2}} \tag{22}$$

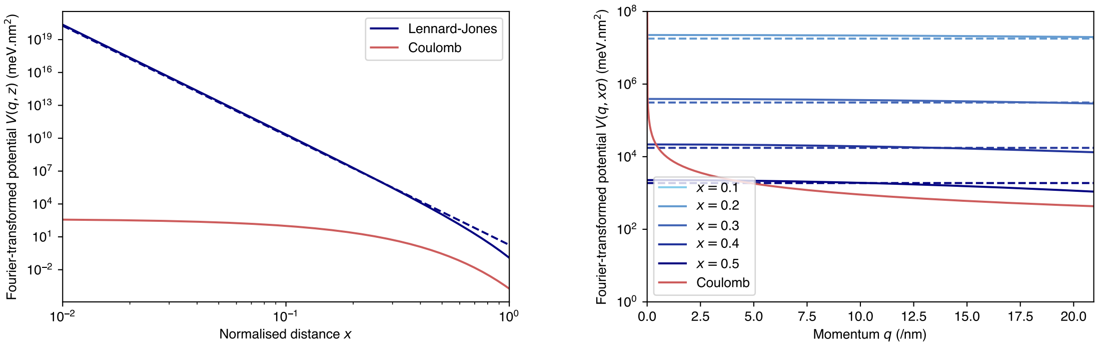

FIG. S6: Fourier-transformed potentials $V(q, z)$ for Lennard-Jones and Coulomb interactions. We used the Lennard-Jones parameters $\mathcal{E}_0 = 0.1$ meV and $\sigma = 0.3$ nm. The dashed lines are the fit $V_{\text{LJ}}(\mathbf{q}, z = \sigma x) \approx (\mathcal{E}_0\sigma^2)/(2x^{10})$. The Coulomb potential is for an elementary charge e, as a function of the normalised distance $x = z/\sigma$ for $q = 10$/nm on **a** and as a function of the momentum q on **b**. The Lennard-Jones uses $z = x\sigma$ and Coulomb uses $z = 0$.

making the $\lambda_{\text{dyn}} \propto \omega_\alpha$ scaling a very general signature of the solid's modes contribution to friction. Still in the limit where solid dynamical modes are well above ω_α, we find the general formula:

$$\boxed{\lambda = \lambda_{\text{stat}} + \lambda_{\text{dyn}} = \frac{\lambda_0}{2}\left(\frac{f_c}{f_\alpha} + \frac{f_\alpha}{f_c}\right)} \tag{23}$$

which we used to fit our experimental results in the main text. Here, we introduce two parameters λ_0 and ω_c which depend on the details of structure factors and the assumed interaction potential. In the following, we use an acoustic phonon branch and a Lennard Jones potential to give an estimate of these parameters in the glycerol-mica case.

4. Phononic friction on mica ?

In order to estimate the friction coefficient, let us consider the case of acoustic phonons in mica – to model the dynamical structure factor $S_{\text{s}}^{\text{dyn}}$ – and Lennard-Jones interactions – to model the interaction potential $V(q, z)$.

a. Lennard-Jones interaction The repulsion between two atoms is modelled by a Lennard-Jones potential

$$V_{\text{LJ}}(\mathbf{r}) = \mathcal{E}_0\left[\left(\frac{\sigma}{r}\right)^{12} - \left(\frac{\sigma}{r}\right)^{6}\right] \tag{24}$$

where $\mathcal{E}_0$ is the interaction energy and σ the typical distance between the atoms. Going to Fourier space,

$$V_{\text{LJ}}(\mathbf{q}, z) = \frac{\pi\mathcal{E}_0\sigma^{10}q^2}{1920z^6}\left[8\left((\sigma q)^2 - 60\left(\frac{z}{\sigma}\right)^4\right)K_2\left(q|z|\right) + q|z|\left(48 + (qz)^2\right)K_3\left(q|z|\right)\right] \tag{25}$$

where K_n is the modified Bessel function of the second kind. This function is plotted in Fig. S6a as a function of the distance $z = x\sigma$. It depends weakly on the wavevector q as plotted in S6b. Thus, a good approximation of the Fourier-transformed Lennard-Jones potential is

$$V_{\text{LJ}}(\mathbf{q}, z = \sigma x) \approx \frac{\mathcal{E}_0\sigma^2}{2x^{10}} \tag{26}$$

In practice, the parameters σ and $\mathcal{E}_0$ and not well known. In the following, we use typical values: $\sigma = 0.3$ nm and $\mathcal{E}_0 = 30$ meV [15].

b. Mica's roughness The mica has a static structure factor related to its atomic roughness. We denote σ_s the inter-atomic distance at the mica surface. Thus, the static structure factor writes

$$S_{\mathrm{s}}^{\mathrm{stat}}(\mathbf{q},\omega) \approx u_s \rho_s \frac{2\pi}{\sigma_s} \delta\left(q - \frac{2\pi}{\sigma_s}\right) \delta(\omega) \tag{27}$$

where $\rho_s \approx 1/\sigma_s^2$ is the atomic density on the interacting layer and u_s is the amplitude of the roughness. Following [16], we expect $u_s \sim 10^{-2}$ for an atomically smooth material. In the following we use $\rho_s = 1/\sigma_{\mathrm{s}}^2$ with $\sigma_{\mathrm{s}} = 0.3$ nm an atomic lengthscale to provide an estimate.

c. Mica's phonons Turning to the excitation modes, we consider acoustic phonons of velocity c in mica. The dispersion relation is $\omega_{\mathrm{ph}}(q) = cq$. Let us compute the associated structure factor in density n using the fluctuation-dissipation theorem. Thus, we compute the response function to an external potential V_{ext}. For this, we return to the classical description of phonons in terms of springs of strength k separating atoms of mass m with a drag coefficient γ and with an inter-atomic distance σ_s. Denoting $\mathbf{u}$ the displacement field, we obtain the dynamical equation

$$m\partial_t^2 \mathbf{u} = -m\gamma\partial_t \mathbf{u} + k\sigma_s^2 \Delta \mathbf{u} - \nabla V_{\mathrm{ext}} \tag{28}$$

In particular, we recognise the phonon speed $c = \sqrt{k\sigma_s^2/m}$. Going to Fourier space and noticing that the density fluctuation is $\delta n = \rho_s \nabla \cdot \mathbf{u}$ where ρ_s is the 2d-particle density, the induced density writes

$$n_{\mathrm{s}}(\mathbf{q},\omega) = \frac{\rho_s q^2/m}{\omega^2 - (qc)^2 - i\gamma\omega} V_{\mathrm{ext}}(\mathbf{q},\omega) \tag{29}$$

Then, taking $\gamma \to 0$, and using the fluctuation-dissipation theorem, we obtain

$$S_{\mathrm{ph}}(\mathbf{q},\omega) = \pi \frac{k_{\mathrm{B}} T \rho_s}{mc^2} \delta(\omega \pm qc) \tag{30}$$

To compare the static and dynamic contributions to friction, there are two main parameters to take into account: the frequency range is different and the the phonons have an effective roughness amplitude. The different frequency range is taken into account by our integration formula Eq.21 and Eq.22. We then use a minimal model to estimate the phonon's spatial amplitude (in units of σ_{s}). Comparing the thermal energy to the phonon mode's energy, we find $u_s^{\mathrm{ph}} = k_{\mathrm{B}}T/(mc^2) \sim 0.1$, which is larger than $u_s \sim 10^{-2}$.

The smallest Young's modulus of mica is estimated at $B = 1$ GPa [12] and its mass density to $\rho = 3 \cdot 10^3$ kg/m^3. We thus estimate the sound velocity as

$$c = \sqrt{\frac{B}{\rho}} \approx 6 \cdot 10^2 \text{ m/s}. \tag{31}$$

Finally, the phonon's wavevector is restricted to the first Brillouin zone, yielding a cut-off at $q_{\max} \sim 2\pi/\sigma_s$.

**Parameter**	**Value**	**Parameter**	**Value**
Inter-atomic distance σ_{s} on mica	3 Å	Atomic density $\rho_s = 1/\sigma_{\mathrm{s}}^2$	10 nm^{-2}
Young modulus of mica B	1GPa [12]	Sound velocity in mica c = $\sqrt{B/\rho}$	600 m/s
Mass density of mica	3.10^3 kg/m^3	Dimensionlaess roughness (in units of σ_{s}) u_s	10^{-2}
Solid's static structure factor $S_{\mathrm{s}}^{\mathrm{stat}}$	–	Liquid structure factor S_l	–
Solid's dynamic structure factor $S_{\mathrm{s}}^{\mathrm{dyn}}$	–	S_{s}'s averaged q-structure $\phi(q)$	?
Lennard Jones energy $\mathcal{E}_0$	30 meV	Glycerol's Debye peak frequency $f_\alpha = \omega_\alpha/2\pi$	–
Phonon mode's spatial amplitude u_s^{ph}	10^{-1}	Experimental critical frequency f_c	34 MHz

Supplementary Table 1: **Table of parameters and definitions for the theoretical model**

d. Friction coefficient The static contribution to the friction coefficient writes:

$$\lambda_{\mathrm{stat}} = \frac{u_s \rho_\ell \rho_s}{8\pi^2 k_{\mathrm{B}} T \omega_\alpha(T)} \left(\frac{2\pi}{\sigma_s}\right)^4 V_{\mathrm{LJ}}\left(\frac{2\pi}{\sigma_s}, z\right)^2 \phi\left(\frac{2\pi}{\sigma_s}\right) \approx \frac{\pi^2 u_s \mathcal{E}_0^2}{2k_{\mathrm{B}} T \omega_\alpha(T) x^{20}} \frac{\rho_\ell \sigma^4}{\sigma_s^6} \phi\left(\frac{2\pi}{\sigma_s}\right) \tag{32}$$

Introducing the Brillouin-zone-averaged q-structure $\bar{\phi}$, we write the dynamic contribution to the friction coefficient:

$$\lambda_{\mathrm{dyn}} = \frac{\rho_\ell \rho_s \omega_\alpha(T)}{8\pi mc^4} \int_0^{q_{\max}} \mathrm{d}q\, q V_{\mathrm{LJ}}(q,z)^2 \phi(q) \approx \frac{\pi^2 \mathcal{E}_0^2 \omega_\alpha(T)}{8mc^4 x^{20}} \frac{\rho_\ell \sigma^4}{\sigma_s^4} \bar{\phi} \tag{33}$$

where we have used that $cq_{\max} \gg \omega_\alpha(T)$.

Summing up these expressions, the total friction writes:

$$\lambda = \lambda_{\rm stat} + \lambda_{\rm dyn} = \frac{\lambda_0}{2}\left(\frac{f_c}{f_\alpha} + \frac{f_\alpha}{f_c}\right) \tag{34}$$

in excellent qualitative agreement with the experimental results (see fit with this formula in Figure 4). We indeed revover the scalings $1/\omega_\alpha$ and ω_α for the static and dynamic contributions to friction at respectively low and high relaxation rates. Eq.34 has two parameters: λ_0 controls the magnitude of the friction coefficient at its minimum, where $\omega = \omega_c$, the crossover between the static and dynamic regimes.

From the liquid and solid structure factors, this crossover frequency writes :

$$\boxed{f_c \approx \frac{1}{2\pi}\sqrt{\frac{4mc^4 u_s}{k_{\rm B}T\sigma_s^2}} \times \sqrt{\frac{\phi\left(2\pi/\sigma_s\right)}{\bar{\phi}}} = 18 \text{ GHz} \times \sqrt{\frac{\phi\left(2\pi/\sigma_s\right)}{\bar{\phi}}}} \tag{35}$$

and λ_0 is the minimal friction coefficient achieved at the critical frequency:

$$\boxed{\lambda_0 \approx \frac{\pi^2\mathcal{E}_0^2}{4c^2x^{20}}\frac{\rho_\ell\sigma^4}{\sigma_s^5}\sqrt{\bar{\phi}\phi\left(\frac{2\pi}{\sigma_s}\right)\frac{u_s}{mk_{\rm B}T}}} \tag{36}$$

We recall that σ, x and $\mathcal{E}_0$ come from the Lennard-Jones potential (*cf. supra*) while σ_s is mica's lattice parameter.

With the phonon model presented above, we obtain expressions of λ_0 and f_c that can be compared to experiments. Experimentally, we find $\lambda_0^{\rm exp}$ = 25.6 MPa.s/m and $f_c^{\rm exp}$ =33.9 MHz. Considering Eq.36, we observe a dramatic dependency on a microscopic parameter $x = z/\sigma$ denoting the effective distance between liquid and wall's molecules. This parameter is key to estimate the interaction potential $V(q)$ and is adjusted at $z \approx 0.18$ nm to match the experimental value of λ_0, a realistic value for such a liquid-solid interface. We now turn to the crossover frequency f_c which interestingly does not depend on the details of the interaction potential (*cf.* Eq.35). We find that f_c is of the order of 18 GHz times a corrective term determined by the spatial dispersion of liquid modes. To account for the experimentally measured $f_c^{\rm exp}$ =33.9 MHz, we need a drastic reduction of ϕ at largest wavevectors. It seems reasonable that modes with wavevectors corresponding to intramolecular movement could see a drastic reduction in their amplitude. Unfortunately, as the q-structure is unknown for glycerol, especially at high frequency, it is hard to push the comparison further.

SUPPLEMENTARY REFERENCES

[1] N.-S. Cheng, Industrial & engineering chemistry research **47**, 3285 (2008).
[2] A. Barlow, A. Erginsav, and J. Lamb, Proceedings of the Royal Society of London. Series A. Mathematical and Physical Sciences **298**, 481 (1967).
[3] D. B. Davies, A. J. Matheson, and G. M. Glover, Journal of the Chemical Society, Faraday Transactions 2: Molecular and Chemical Physics **69**, 305 (1973).
[4] G. Harrison, *The dynamic properties of supercooled liquids* (1976).
[5] M. H. Jensen, C. Gainaru, C. Alba-Simionesco, T. Hecksher, and K. Niss, Physical Chemistry Chemical Physics **20**, 1716 (2018).
[6] P. Lunkenheimer, U. Schneider, R. Brand, and A. Loid, Contemporary Physics **41**, 15 (2000).
[7] D. W. Davidson and R. H. Cole, The Journal of Chemical Physics **19**, 1484 (1951).
[8] P. Lunkenheimer, A. Pimenov, B. Schiener, R. Bohmer, and A. Loidl, Europhys. Lett. **33**, 611 (1996).
[9] O. I. Vinogradova, Langmuir **11**, 2213 (1995).
[10] C. Cottin-Bizonne, A. Steinberger, B. Cross, O. Raccurt, and E. Charlaix, Langmuir **24**, 1165 (2008).
[11] S. Leroy and E. Charlaix, J. Fluid Mech. **674**, 389 (2011-05-10).
[12] L. McNeil and M. Grimsditch, Journal of Physics: Condensed Matter **5**, 1681 (1993).
[13] Z. Zhang, V. Bertin, M. H. Essink, H. Zhang, N. Fares, Z. Shen, T. Bickel, T. Salez, and A. Maali, Journal of Fluid Mechanics **977**, A21 (2023).

[14] L. Bocquet and J.-L. Barrat, , 9 (2007).
[15] D. A. Jahn, F. O. Akinkunmi, and N. Giovambattista, The Journal of Physical Chemistry B **118**, 11284 (2014).
[16] L. Bocquet and J.-L. Barrat, Phys. Rev. E **49**, 3079 (1994).
